# Fluorescein angiographic findings and Behcet's disease ocular attack score 24 (BOS24) as prognostic factors for visual outcome in patients with ocular Behcet's disease

**DOI:** 10.1186/s40942-021-00318-4

**Published:** 2021-08-28

**Authors:** Narumon Keorochana, Nathamon Homchampa, Sritatath Vongkulsiri, Raveewan Choontanom

**Affiliations:** 1 Ocular Inflammatory and Uveitis Division, Department of Ophthalmology, Phramongkutklao Hospital, 315 Ratchvidhi Rd. Thung Phayathai, Ratchathewi, Bangkok, 10400 Thailand; 2 Department of Ophthalmology, Phramongkutklao Hospital, 315 Ratchvidhi Rd. Thung Phayathai, Ratchathewi, Bangkok, 10400 Thailand; 3 Retina Division, Department of Ophthalmology, Phramongkutklao Hospital, 315 Rajawithi Rd, Thung Phayathai, Ratchathewi, Bangkok, 10400 Thailand; 4 Oculoplastic and Reconstructive Surgery Division, Department of Ophthalmology, Phramongkutklao Hospital, 315 Rajawithi Rd, Thung Phayathai, Ratchathewi, Bangkok, 10400 Thailand

**Keywords:** Fluorescein Angiography, BOS24, Behcet's disease, Visual prognosis, Ocular Behcet

## Abstract

**Purpose:**

To determine the application of fluorescein angiographic (FA) findings and Behcet’s disease ocular attack score 24 (BOS24) scoring system in predicting poor visual outcome in patients with ocular Behcet’s disease.

**Study design:**

Retrospective cohort study.

**Methods:**

We included 73 eyes of 38 patients with ocular Behcet’s disease who underwent FA and reviewed FA images, anterior chamber cells, vitreous opacity, retinal and optic disc lesions, which are parameters in BOS24. The correlation between FA findings, BOS24, and visual acuity was assessed.

**Results:**

Optic disc hyperfluoresence (74%), diffuse posterior pole leakage (52%) and diffuse peripheral leakage (52%) were the three most common findings. Common complications were peripheral capillary nonperfusion (29%), arterial narrowing (22%), and macular ischemia (19%). BOS24 scores of ≥ 6 (p < 0.0001), arterial narrowing (p < 0.0001), and severe posterior pole leakage (p = 0.004) were significantly associated with poor visual outcome. Combining significant FA findings: arterial narrowing and severe posterior pole leakage, to BOS24 ≥ 6 results in an increased relative risk of developing poor visual acuity from 7.30 to 10.43 and 1.89 to 2.02 respectively.

**Conclusion:**

Fluorescein angiography is an important investigation for predicting poor visual outcome. BOS24 may be a useful alternative when fluorescein angiographic is unavailable.

## Introduction

Behcet’s disease(BD) is a chronic relapsing inflammatory disease of unknown origin. Its pathophysiology is suggested to be caused by an immune-mediating etiology resulting in vasculitis and subsequently damaging blood vessels throughout the body including the retina [1]. Although BD is a multisystem disease, ocular complications are the most devastating conditions and have a large impact on the patient's quality of life [2]. Since the onset of disease starts at a young age, patients will be carrying the visual burden for more than half their life. In the past 30 years [3], despite using the appropriate treatment and intensive follow up, patients with ocular BD still carried a poor visual prognosis [4], with a reported loss of useful vision in 74% of the eyes six to ten years after the onset of the ocular symptoms [5]. In a large international collaborative study with 1465 ocular BD patients from 25 eye centers in 14 countries, 23% of patients had a visual acuity equal to or worse than 20/200 at the final visit [6]. The rates of ocular manifestations and visual prognosis also differ in each country with India, Iran, and Japan having a higher rate of poor vision [6]. This is one of the few studies in Thailand to study the prognostic factors in ocular BD patients.

Although ocular BD is a sight-threatening disease, early detection, effective treatment of uveitis with rapid control of intraocular inflammation, and prevention of further attacks are very important to prevent visual loss in ocular BD patients [1, 2]. According to several recent studies, after the use of immunosuppressive [3] and biological agents [7–13] such as infliximab and adalimumab, the visual prognosis has substantially improved with a 10-year risk of severe visual loss of 13% [7]. An expert panel from the American Academy of Ophthalmology [14] has given a strong recommendation in favor of treatment with anti-TNF therapy with infliximab or adalimumab (moderate-quality evidence) as first-or second-line corticosteroid-sparing agents for patients with ocular BD [6]. But currently, there is no standard treatment or indication of when to initiate therapy with biological agents and of which drug, dosage, and duration. Due to their high cost, availability, and limited experience with these new drugs, they are most often reserved for refractory cases which are already in advanced stages with little potential for improvement, resulting in unsatisfying results [14]. Thus in the future, the ability to predict a patient’s visual prognosis will be very important, as eligible patients may be good candidates for initiating biological agents or at best receive the most appropriate treatment available to preserve maximum vision.

The risk and prognostic factors for visual outcome might be greatly influenced by the severity of the disease. Previous studies have tried to determine risk factors for predicting poor visual outcome in ocular BD using initial BCVA [15, 16], the frequency of attacks, duration of uveitis [2], and location of vasculitis [17]. Fluorescein Angiography (FA) is currently the gold standard investigation in BD as it is essential for revealing the severity, location, and extent of the disease [18]. FA Findings have also been used to helpfully predict visual prognosis in ocular BD [17, 19]. Especially, ultra-widefield retinal imaging is still very useful to evaluate better quantify the extent of the inflammation [1]. Posterior pole involvement, the degree of vascular leakage, optic disc hyperfluorescence, macular leakage [17], macular ischemia, Neovascularization at disc (NVD) [19] arterial narrowing [20], and dense vitreitis [8] were significantly associated with worse vision but results have been variable between studies. Though beneficial, FA is invasive and is not available in every hospital. Moreover, due to its limitations and contraindications, it may not be performed in every ocular BD patient. Recently, a new novel scoring system for determining the activity of ocular BD termed Behçet’s disease ocular attack score 24 (BOS24) [21–23] has been used to predict VA deterioration and its results have been promising. Nevertheless, different tools used in predicting visual prognosis have different advantages and different limitations. This study aims to assess the application of fluorescein angiographic (FA) findings and the BOS24 scoring system in predicting poor visual outcomes in patients with ocular BD.

## Material and methods

### Patient selection

Medical records of ocular BD patients diagnosed and treated at Phramongkutklao Hospital from January 2001 to January 2018 were retrospectively reviewed. This study was approved by the Institutional Review Board, Royal Thai Army Medical Department (IRB RTA) and the protocol adhered to the Declaration of Helsinki.

Our study included patients with ocular BD who was diagnosed with Behcet's disease based on The International Criteria for Behçet’s Disease (ICBD) [24] and had previous FA results in the Hospital's database. Every available FA in each patient was studied for signs of retinal vasculitis and other complications. Patients with HIV infection, active co-infections, tuberculosis, sarcoidosis, diabetic retinopathy, age-related macular degeneration, hypertensive retinopathy, dense cataract, glaucoma, previous intraocular surgery, severe vitreous opacity and low-quality FA were excluded from the study.

### Data collection

The demographic data gathered were gender, age at the time of diagnosis, and laterality. For clinical records, each patient underwent a complete ophthalmic examination by the same uveitis specialist. Their medical records and ocular findings were reviewed at each visit including best-corrected visual acuity (BCVA) by ETDRS chart which was then converted into the logarithm of the minimal angle of resolution (logMAR), anterior and posterior segment findings, the number of attacks, duration of follow up and the treatment regimen were recorded. For every attack, the BOS24 was determined for each patient (Table 1) which consists of 24 points summarized from six ocular parameters including anterior chamber cells (maximum 4 points), vitreous opacity (maximum 4 points), peripheral fundus lesions (maximum 8 points), posterior pole lesions (maximum 4 points), subfoveal lesions (maximum 2 points) and optic disc lesions (maximum 2 points). BOS24-2YR was calculated in all available patients and for the analysis of individual BOS24 scores, the Maximum BOS24 was used.

**Table 1:** Reprinted from Behcet’s disease ocular attack score 24: evaluation of ocular disease activity before and after initiation of infliximab by Toshikatsu Kaburaki et al.

BOS24 [22]
(1) Cells in the anterior chamber (max. 4 points)
Cell 0: 0 point, cell 0.5+ or 1+ : 1 point, cell 2+ : 2 points, cell 3+ : 3 points, cell 4+ or hypopyon: 4 points
(2) Vitreous haze (max. 4 points) Haze 0: 0 point, haze 0.5+ or 1+ : 1 point, haze 2+ : 2 points, haze 3+ : 3 points, haze 4+ : 4 points
(3) New inflammatory changes in the peripheral retina (max. 8 points)
Give each 2 points in each quadrant of peripheral retina if new inflammatory changes (exudates, hemorrhages, vasculitis) are seen
(4) New inflammatory changes in the posterior pole of retina (max. 4 points)
The percentage of areas occupying new inflammatory changes in the posterior pole of retina:
0%: 0 point, > 0 and < 10%: 2 points, ≥ 10 and < 25%: 3 points, ≥ 25%: 4 points
(5) New inflammatory changes in the fovea (max. 2 points)
Give 2 points if new inflammatory changes (exudates, hemorrhages, vasculitis) are seen in the fovea
(6) New inflammatory changes in the optic disc (max. 2 points)
Give 2 points if new inflammatory changes in the optic disc (redness and edema, sometimes accompanied by hemorrhages, exudates and edema of retina surrounding the optic disc) are seen

### Fluorescein angiography

Images were obtained from Spectralis™ (Heidelberg Engineering, Heidelberg, Germany) and Kowa Fundus Camera model VX-10i (Kowa, Japan). Multiple Images were selected from early-phase and late-phase FA images, and interpreted by an experienced retinal specialist (SV) and an experienced uveitis specialist (NK). Both were blinded from the patient's clinical information and the other’s reading. In cases of disagreement, FA revision was done with both interpreters till a consensus was reached. For patients who underwent serial FAs, all available FAs were included in the study. FAs with poor quality due to media opacity or inadequate dilation were excluded from our study. Angiographic findings that were examined included the location of vasculitis (peripheral, posterior pole, macular and extramacular), the degree of leakage (mild, moderate, severe), and optic disc hyperfluorescence (partial, diffuse with/without blurred disc). Other FA finding present were also recorded such as peripheral capillary nonperfusion, vascular anastomosis, neovascularization elsewhere(NVE), neovascularization at disc (NVD), branch retinal vein occlusion (BRVO), central retinal vein occlusion (CRVO), central retinal artery occlusion (CRAO), cystoid macular edema (CME), macular ischemia, and arterial narrowing.

**Fig. 1:**
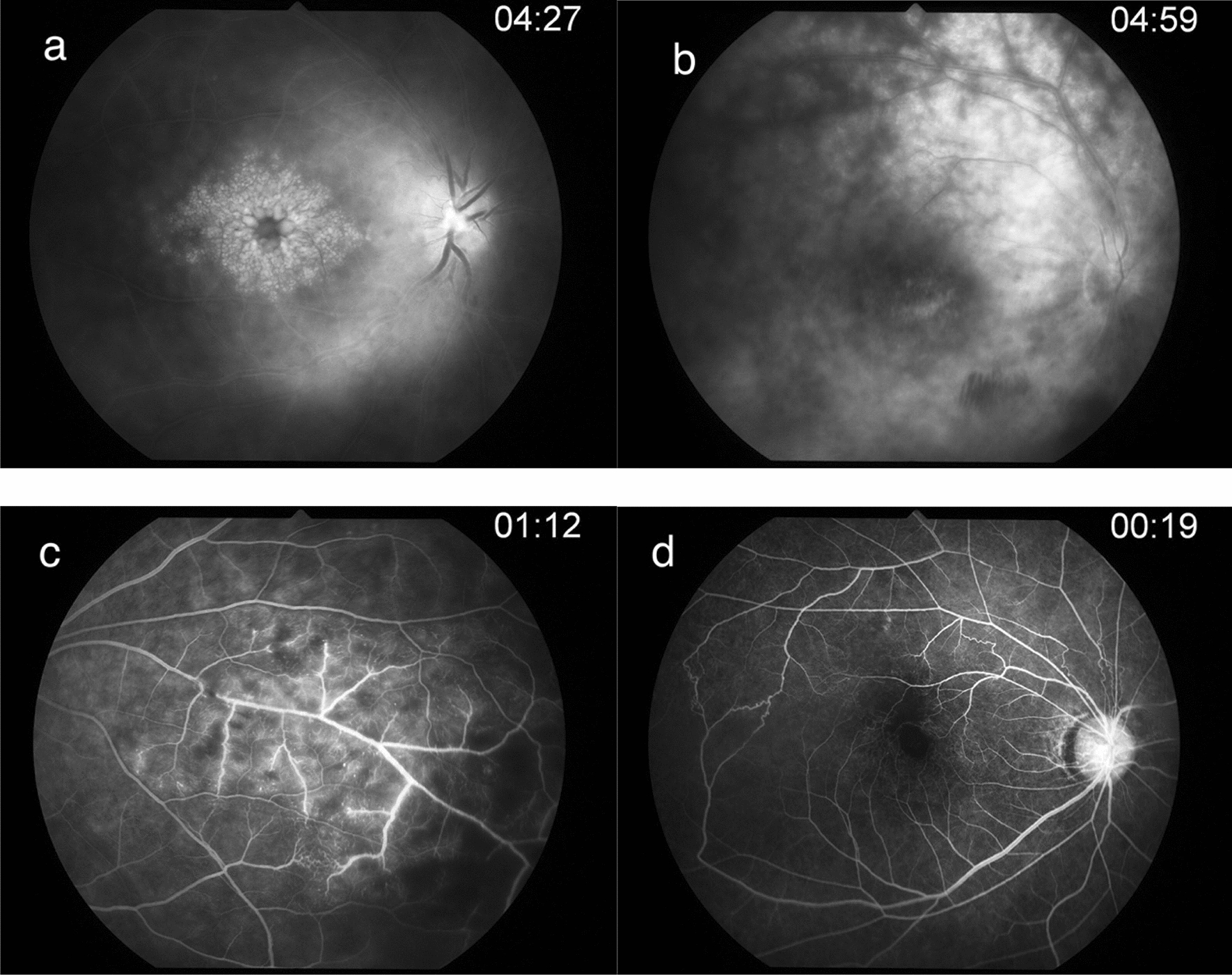
FA images show (**a**). Diffuse severe macular leakage with moderate extramacular leakage and diffuse optic disc hyperfluorescence with blurring of disc margin (**b**). Severe diffuse vascular leakage at the posterior pole and periphery with mild focal perifoveal hyperfluorescence and diffuse optic disc hyperfluorescence with clear disc margin (**c**). Mild focal peripheral vascular leakage with capillary nonperfusion (**d**). Optic disc hyperfluorescence with macular ischemia and vascular anastomosis in early phase of angiography

The grading system used in this study was adapted from criteria used in dual fluorescein and ICG angiographic scoring system for uveitis by the Angiographic Scoring for Uveitis Working Group (ASUWOG) [25]. Eyes were classified based on the anatomical location of retinal vascular leakage. Leakage localized at the posterior pole was classified as posterior pole vasculitis which was further classified into macular and extramacular. While leakage at the periphery was defined as peripheral vasculitis. The extent of leakage was classified as focal if a well-defined area of leakage could be localized in 1–2 quadrants, or diffuse if there were multifocal leakage in more than 2 quadrants. The degree of severity was graded using late phases images and graded as mild for minimal staining and leakage, moderate for a greater leakage with well-defined margins, and severe for massive leakage with the blurring of vessel margins. Optic disc hyperfluorescence was graded in the late phases defined as none for normal late staining, partial for partial staining at disc, diffuse for diffuse disc leakage which was further categorized into diffuse with clear disc margin, or diffuse with the blurring of the disc margin (Fig. 1).

### Statistical analysis

Gender, age, number of attacks, duration of follow-up, ETDRS visual acuity values (converted to logMAR) BOS24 scores, FA findings were recorded for each patient. The median of baseline logMAR VA was compared by the Mann–Whitney U test according to the presence or absence of each FA characteristic. Mean BCVA of the first presentation and last BCVA was compared using Wilcoxon rank T-test. FA findings were compared between non-legally blind (VA better than 20/200) patients and legally blind patients (VA worse or equal to 20/200) using the chi-square test. The FA characteristics and latest Best-corrected visual acuity (BCVA) were analyzed using the Pearson chi-square test (Fisher’s exact test was used when expected cell counts were less than five). All significant findings from the previous test were reanalyzed using multivariable linear regression analysis. The Final BCVA was used as the dependent variable and all significant FA findings as independent variables. A p-value of less than 0.05 was considered significant. IBM SPSS statistics software (ver.25; SPSS Science, Chicago, IL, USA) was used for all statistical analyses.

## Results

Fifty-two patients with ocular BD were seen at Phramongkutklao Hospital during the study period; 14 patients were excluded from the study due to unavailable FA results and incomplete medical records. A total of 73 eyes from 38 patients were included in our study (Table 2). The mean age of patients was 38.3 ± 9.5 (21–54 years), the mean duration of follow-up were 5.4 ± 4.3 years (8 months to 17 years) and the number of attacks was 2.1 ± 1.9 attacks. All of which were not significantly related to poor visual acuity with P-values of 0.98, 0.091, and 0.336 respectively. The majority of patients were males, with a total of 29 (76.3%) and a mean age of 38.3 ± 9.8 years. There were 9 (23.7%) females in the study with a mean age of 38.3 ± 9.2 (P = 0.98). Mean visual acuity at first presentation and last follow-ups were 0.7 ± 0.8 and 0.7 ± 0.9 (P = 0.665). The presence of each ocular parameter in BOS24 was not related to poor VA in univariate analysis (p > 0.05). However, the vitreous haze was statistically significantly related after multivariate analysis (p = 0.011). Thirty-seven (97.4%) patients received immunosuppressants including cyclosporine, methotrexate, azathioprine, mycophenolate mofetil and cyclophosphamide. There were 25 (67.6%) patients who received 1 drug and 12 (32.4%) patients who received 2 drugs. Nobody in this study received biologics or Interferon-alpha (IFN alpha).

**Table 2:** Baseline characteristics

	Mean ± SD (%)		
Total of patients (38)			
Male	29 (76.3)		
Female	9 (23.7)		
Male: female	3.2: 1		
Age (years)	38.3 ± 9.5	21–54	P = 0.98
Bilateral	35 (92.1)		
Total of eyes (73)			
BCVA at presentation	0.7 ± 0.8	0–2.7	P < 0.001*
BCVA at last visit	0.7 ± 0.9	0–4.7	
Frequency of attacks	2.1 ± 1.9	1–9	P = 0.336
Duration of Follow up (years)	5.4 ± 4.2	8 mo–17 y	P = 0.091
BOS24-2YR	7.1 ± 7.4	0–33	P < 0.001*
BOS24 (Max)	4.8 ± 4.9	0–22	P < 0.001*
Anterior chamber cells	0.6 ± 0.9	0–4	P > 0.05
Vitreous opacity	1.0 ± 1.0	0–4	P > 0.05
Peripheral fundus lesions	1.3 ± 2.2	0–8	P > 0.05
Posterior pole lesions	0.9 ± 1.3	0–4	P > 0.05
Subfoveal lesions	0.6 ± 0.9	0–2	P > 0.05
Optic disc lesions	0.6 ± 0.9	0–2	P > 0.05

^*^P-value < 0.05, Independent sample test, Wilcoxon rank T-test

FA was available in 73 eyes (Table 3). Vasculitis found in 55 eyes (75. 3%). Categorized by location, 47 patients had Posterior Pole leakage (64.4%), 44 patients had Peripheral leakage (60.3%) and 36 patients had Peripheral & Posterior pole leakage (49.3%). As for the extent of leakage diffuse posterior pole vasculitis was found in 38 patients (52.1%), diffuse peripheral vasculitis in 31 patients (42.5%) and diffuse macular vasculitis in 20 patients (27.4%). Very few patients had focal leakage (Focal peripheral leakage 5.5%, Focal posterior pole leakage 9.6%). Optic disc hyperfluorescence found in 75.3%, with the majority of 35.6% with diffuse with blurring of disc margin followed by 21.9% with diffuse with clear disc margin and 17.8% with partial disc hyperfluorescence. The most common ischemic changes found on FA were peripheral capillary nonperfusion (28.8%), arterial narrowing (21.9%), and Macular ischemia (19.2%). Table 4 compares the median baseline logMAR VA of eyes with and without FA characteristics. The baseline logMAR VA for eyes with vascular anastomosis (p = 0.03), macular ischemia (p ≤ 0.0001), CRAO (p = 0.037), and arterial narrowing (p = 0.00) was significantly worse compared to eyes without the following findings. At the end of the study, nineteen eyes (26%) had poor visual acuity, defined as having BCVA ≤ 20/200. Univariable linear regression analysis was done and identified that leakage at the posterior pole, leakage at the posterior pole and periphery, moderate posterior pole leakage, severe posterior pole leakage, diffuse posterior pole leakage, vascular anastomosis, macular ischemia, arterial narrowing were factors that were significantly associated with final poor visual acuity (all p < 0.04) (Table 5). But after multivariable regression analysis, only arterial narrowing (P < 0.0001, RR = 7.3, 95% CI 1.99–26.75) and severe posterior pole leakage (p = 0.041, RR = 1.88 95% CI 1.07–3.33) remained significant risk factors (adjusted R2 = 0.35) (Table 5).

**Table 3:** Fluorescein Angiography Findings in ocular Behcet’s disease

FA characteristics	N	P value	RR	95% CI
Peripheral leakage	44 (60.3%)	0.062		
Posterior Pole leakage	47 (64.4%)	0.011*	1.446	1.135–1.842
Peripheral & Posterior pole leakage	36 (49.3%)	0.013*	1.415	1.059–1.891
Diffuse peripheral vasculitis	31 (42.5%)	0.008*	1.476	1.068–2.040
Diffuse macular vasculitis	20 (27.4%)	0.135		
Diffuse posterior pole vasculitis	38 (52.1%)	0.008*	1.463	1.103–1.942
Degree of retinal vascular leakage				
Mild peripheral vasculitis	18 (24.7%)	0.672		
Moderate peripheral vasculitis	20 (27.4%)	0.135		
Severe peripheral vasculitis	7 (9.6%)	0.196		
Mild posterior pole leakage	14 (19.2%)	1.0		
Moderate posterior pole leakage	17 (23.3%)	0.024*	1.518	0.952–2.42
Severe posterior pole leakage	16 (21.9%)	0.004*	1.885	1.068–3.327
Optic disc hyperfluoresence	54 (74%)	0.126		
Partial	13 (17.8%)			
Diffuse with clear disc margin	16 (21.9%)			
Diffuse with blurring of disc margin	25 (34.3%)			
Peripheral capillary nonperfusion	21 (28.8%)	0.557		
Vascular anastomosis	12 (16.4%)	0.038*	1.574	1.003–2.813
Neovascularization elsewhere	7 (9.4%)	0.668		
Neovascularization at disc	4 (5.5%)	1.0		
Branch retinal vein occlusion	2 (2.7%)	0.455		
Central retinal vein occlusion	3 (4.1%)	0.164		
Central retinal artery occlusion	2 (2.7%)	0.065		
Cystoid macular edema	8 (11.0%)	0.102		
Macular ischemia	14 (19.2%)	< 0.001*	2.325	1.141–4.740
Arterial narrowing	16 (21.9%)	< 0.001*	7.298	1.991–26.750

^*^P-value < 0.05, Pearson Chi-square test

**Table 4:** Comparison of best corrected visual acuity at FA between the eyes: with and without each angiographic characteristic

	LogMARmedian ± IQR		P-Value
	absent	present	
Peripheral involvement	0.10 ± 0.48	0.48 ± 1.11	0.002*
Isolated peripheral involvement	0.30 ± 0.90	0.50 ± 0.64	0.866
Peripheral and posterior involvement	0.14 ± 0.63	0.48 ± 1.12	0.007*
Posterior pole involvement	0.10 ± 0.48	0.48 ± 0.82	< 0.001*
Diffuse posterior pole leakage	0.10 ± 0.60	0.48 ± 1.84	0.003*
Severe Posterior pole leakage	0.18 ± 0.50	1.15 ± 1.82	< 0.001*
Peripheral capillary nonperfusion	0.3 ± 0.98	0.4 ± 0.73	0.280
Vascular anastomosis	0.18 ± 0.64	0.94 ± 0.6	0.03*
Neovascularization elsewhere	0.4 ± 0.9	0.2 ± 0.38	0.992
Neovascularization at disc	0.4 ± 0.9	0.39 ± 1.69	0.632
Branch retinal vein occlusion	0.4 ± 0.9	1.05 ± ^a^	0.731
Central retinal vein occlusion	0.35 ± 0.81	2.3 ± ^a^	0.326
Central retinal artery occlusion	0.3 ± 0.78	2.15 ± ^a^	0.037*
Cystoid macular edema	0.4 ± 0.9	0.29 ± 0.36	0.600
Macular ischemia	0.18 ± 0.5	1.0 ± 1.45	< 0.001*
Arterial narrowing	0.18 ± 0.38	1.65 ± 1.3	< 0.001*

^a^IQR not calculated due to insufficient data

^*^P-value < 0.05, Mann–Whitney U test

**Table 5:** Multivariate logistic regression analysis of the risk factors for poor visual acuity (≤ 20/200) among FA characteristic

	Unstandardized coefficients	Std. error	Standardized coefficients	t	Sig
	Beta		Beta		
Anterior chamber cells	− 0.092	0.137	−0.089	− 0.674	0.503
Vitreous haze	0.300	0.114	0.334	2.632	0.011*
Posterior pole leakage	− 0.052	0.276	− 0.028	− 0.188	0.852
Diffuse Posterior pole leakage	0.157	0.215	0.090	0.733	0.466
Moderate posterior pole leakage	0.354	0.239	0.170	1.483	0.143
Severe Posterior Pole Leakage	0.756	0.242	0.356	3.127	0.003*
Peripheral and posterior pole leakage	− 0.132	0.238	− 0.075	− 0.555	0.581
Arterial narrowing	0.643	0.239	0.303	2.684	0.009*
Macular ischemia	0.227	0.272	0.102	0.834	0.408
Vascular anastomosis	− 0.252	0.265	− 0.106	− 0.949	0.346

^*^P-value < 0.05, multivariable linear regression analysis

**Fig. 2:**
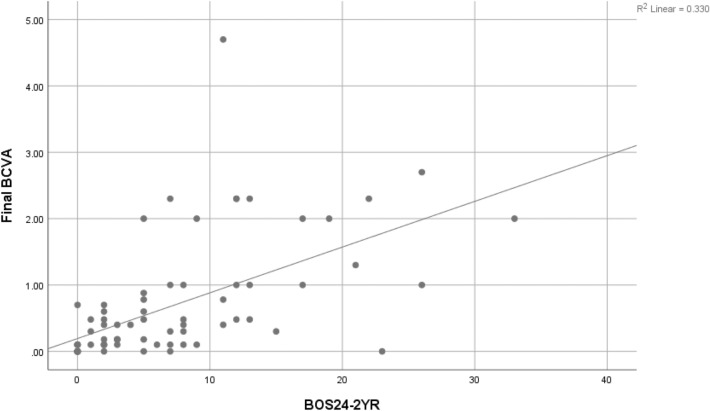
Association between Final BCVA and BOS24-2YR, (linear approximate equation: y = 0.19 + 0.07 × R^2^ = 0.330; r(58) = 0.574, p < 0.0001)

**Fig. 3:**
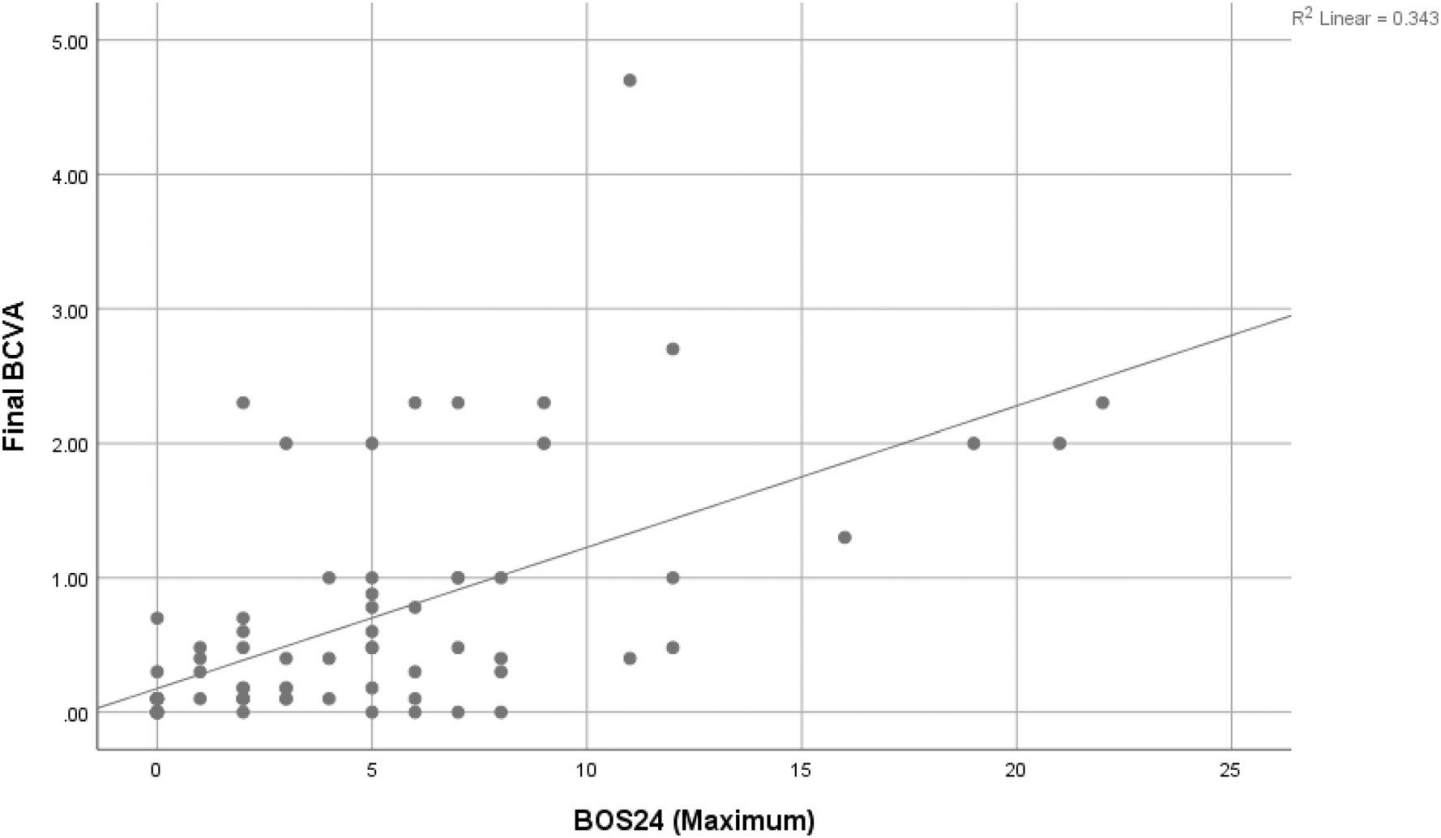
Association between Final BCVA and BOS24 (Maximum), (linear approximate equation: y = 0.18 + 0.11 × R^2^ = 0.343; r(71) = 0.586, p < 0.0001). Study conception and design: Keorochana N, Homchampa N. Acquisition of data: Homchampa N, Vongkulsiri S. Analysis and interpretation of data: Keorochana N, Homchampa N. Drafting of manuscript: Keorochana N, Homchampa N, Vongkulsiri S. Critical revision: Keorochana N, Choontanom R

As for BOS24 scores, two analyses were made. First, the BOS24-2YR was calculated in available patients (N = 30) and resulted in a significant positive relationship between the BOS24-2YR score and poor visual acuity (VA ≤ 20/200) r(58) = 0.58, p < 0.0001 (Fig. 2). Individual BOS24 scores also had a significant positive correlation with poor VA r(71) = 0.59, p < 0.0001 (Fig. 3). The maximum BOS24 scores of each patient were used to create a ROC curve. Using a cut-point of BOS24 ≥ 6 per attack, it resulted in a sensitivity of 73.7% (95% CI 48.80% to 90.85%) and a specificity of 79.6% (95% CI 66.47% to 89.37%) in predicting poor visual outcome. Further univariable linear regression was done and was also found to be statistically significant p < 0.0001, RR = 2.0, 95% CI (1.295 to 3.201). When BOS24 ≥ 6 was used in combination with significant FA findings (arterial narrowing & severe posterior pole leakage), it still produced significant results with P-values of < 0.0001 and 0.025 respectively and increased the relative risks of developing poor visual outcomes from 7.3 to 10.4 when combined with arterial narrowing and from 1.9 to 2.0 when combined with severe posterior pole leakage (Table 6).

**Table 6:** Prognostic factors for poor visual outcome

Relative risk		95% CI	Sensitivity (%)	Specificity (%)	p-value
BOS24 ≥ 6	2.0	1.295–3.201	73.7	79.6	< 0.001*
Arterial narrowing	7.3	1.991–26.750	73.7	96.3	< 0.001*
Severe posterior pole leakage	1.9	1.068–3.327	36.8	85.2	0.004*
BOS24 ≥ 6 & Arterial narrowing	10.4	1.592–68.264	57.9	98.2	< 0.001*
BOS24 ≥ 6 & Severe posterior pole leakage	2.0	1.256–3.261	26.3	94.4	< 0.001*

^*^P-value < 0.05, univariable linear regression

## Discussion

Behcet’s disease is a chronic, relapsing, multisystem inflammatory disorder with sight-threatening complications occurring mostly in the working-age group. Due to the introduction of biological agents, the visual prognosis of ocular BD patients has improved since 1990 [3, 26]. Predicting visual prognosis is essential for appropriate treatment and follow-up and in the near future, when biological agents are more widely available, may be used as an indicator of such treatment.

This is the first study to assess both FA Findings and BOS24 in terms of predicting visual acuity. Earlier studies [21, 22, 27] have assessed BOS24 as a range over a target period that is not applicable to everyday clinical use. We intended to examine the use of BOS24 as individual attacks which might be more useful in practical clinical settings and also determine FA findings that are associated with poor visual acuity.

In our study, BOS24 scores of ≥ 6, severe posterior pole leakage, and arterial narrowing were significantly associated with poor visual outcomes. In a recent study by Rie Tanaka et al. [22] BOS24-5Y was a significant positive prognostic index for poor visual prognosis in patients with ocular BD. We also analyzed BOS24-2YR and its correlation with poor visual acuity (VA ≤ 20/200) and found that it was statistically significant which was consistent with the previous study. (r(58) = 0.574, p < 0.0001) Further analysis was done with individual BOS24 scores and a cut point of BOS24 ≥ 6 was significantly associated with poor visual outcome (RR = 2.036, 95% CI 1.295–3.201, sensitivity 73.7%, specificity 79.6% p-value < 0.0001) But in Rie Tanaka et al.‘s [22] study posterior segment lesions were found to be significantly associated with visual change but in this present study after multivariate regression analysis instead vitreous haze was associated with poor final visual outcome. The discrepancies might be due to the different inclusion criteria since the study by Rie Tanaka, et al. [22] included patients with BCVA of logMAR < 1.0, many patients with poor VA due to some degree of vitreous haze might have been excluded. The study by Masaru Takeuchi et al. [26] about risk and prognostic factors of poor visual outcome in Behcet’s disease with ocular involvement also stated that severe vitreous opacity were prognostic factors for poor visual outcome as strong vitreous opacity, though temporary and reversible, are mainly caused by severe inflammatory retinal lesions in which the neural retina can be damaged by abundant inflammatory products causing permanent damage and eventually visual loss. Results of severe posterior pole leakage were also consistent with previous studies [17, 28] suggesting that lesions within the posterior pole were more likely to be associated with irreversible visual loss due to damage to photoreceptors and ganglion cells in the macula by inflammatory cell-mediated products. Fatemeh Bazvand et al. [20] suggested that arterial narrowing on FA could predict poor visual prognosis which is consistent with our study. As arterial narrowing is a sign of end-stage disease from multiple episodes of vasculitis or from complications such as retinal artery or vein occlusion which explains why it is highly associated with poor visual outcomes. Though it is a very significant factor for poor visual prognosis (RR = 7.298), it is an irreversible condition. Even after aggressive treatment, substantial visual improvement is unlikely to occur.

Male predominance has frequently been demonstrated in nearly every previous study [6]. Our study has a male predominance of 3.2:1 which is similar to the previously reported ratios [2, 19]. In a recent study Min Kim [17] found that the mean VA of patients with posterior pole vasculitis was significantly worse compared to patients with peripheral vasculitis but this wasn’t observed in our study. This might be because most patients with peripheral vasculitis also had concurrent posterior pole vasculitis with only 8 patients (11%) with pure peripheral vasculitis. Optic disc hyperfluorescence has also been suggested as being a marker of disease activity and Gedik et al. [18] had reported optic disc leakage/staining in 89.8% of eyes which was fairly similar to our study of 74%. But we found no significant relationship between the amount of disc leakage and VA in the present study. Macular leakage which was expected to be significantly associated with poor visual acuity was not significant in our study. These results were consistent with Nussenblatt et al. [29] which found that there was no significant relationship between visual acuity and the amount of fluorescein staining measured in the posterior pole but visual acuity was instead significantly related with macular thickening on Optical coherence tomography (OCT) which was not done in our cohort. In addition, this can also be explained by the very low number of patients with pure macular leakage (N = 3), as most of the patients with macular leakage also had other lesions in the posterior pole. However, fluorescein leakage doesn't always indicate active vasculitis as after recurrent attacks chronic damage to the endothelial blood-retinal barrier can result in persistent fluorescein leakage. This explains the lack of correlation between mild vascular leakage and visual acuity in our study. An adjunctive use of BOS24 to indicate disease activity might help distinguish active inflammation and chronic persistent leakage.

Despite the simpleness and usefulness of BOS24, it is not equivalent to FA. Hormoz Chams, et al. [30] had studied BD patients without apparent ocular signs and VA of 20/20 in both eyes in which BOS24 scores would have been zero. Forty-four percent of these patients revealed vascular leakage on FA, mostly at the periphery. Therefore, FA is essential for the early detection of ocular BD without obvious ocular signs.

Macular ischemia and NVD, both ischemic complications were reported in many studies [16, 19, 31, 32] as poor prognostic factors for visual acuity. However, even though they were not found to be significant in our cohort, we still believe that both are associated with poor visual acuity as they are caused by extensive retinal nonperfusion [33]. Macular ischemia was a significant factor after univariate analysis but not significant after adjusting other factors. As for NVD, the number of patients with NVD (N = 4) might have been too little to produce a significant effect. Having a presence of vascular anastomosis suggests that the patient might have had cumulative ischemic damage for a period of time presumably from vaso-occlusive inflammation, though not significantly associated with poor VA after multivariate regression, we suggest that patients will have a tendency to develop poor visual acuity.

Our study is a retrospective cohort which Inevitably contains bias and limitations. Firstly, our hospital is a tertiary care center with the tendency of more severe cases referred from other hospitals beyond their capabilities of care. It might not represent the natural pattern of the disease. The number of patients included in the study was similar to many studies but was still too small to detect minor differences in FA findings. Patients included in the study had different durations of follow-up varying from 8 months to 17 years, so each patient's final BCVA is evaluated at a different point in time. BCVA was evaluated mostly by pinhole which might not represent the true VA in every patient. As each patient varied in disease severity, they were treated with different drugs which might have had an effect on visual outcome. We modified a previously used criterion in grading FA characteristics and even though we used two experienced ophthalmologists there might still be subjectivity in grading leakages and FA findings. Further prospective studies with more patients, longer follow up and the same treatment regimen is warranted.

## Conclusion

Fluorescein angiography is an important investigation for predicting poor visual outcome. Therefore it should be done in every patient. However, BOS24 may also be a useful alternative when FA is unavailable, such as the limitation of time, cost or machine.

## Data Availability

For data availability, please contact corresponding author for data requests.

## Abbreviations

FA: Fluorescein angiography
BD: Behcet’s disease
BOS24: Behcet’s disease ocular attack score 24
BCVA: Best-corrected visual acuity
NVE: Neovascularization elsewhere
NVD: Neovascularization at disc
BRVO: Branch retinal vein occlusion
CRVO: Central retinal vein occlusion
CRAO: Central retinal artery occlusion
CME: Cystoid macular edema
OCT: Optical coherence tomography
